# High Weight-Specific Power Density of Thin-Film Amorphous Silicon Solar Cells on Graphene Papers

**DOI:** 10.1186/s11671-019-3132-6

**Published:** 2019-10-16

**Authors:** Xin Zhang, Chi Zhang, Dongdong Li, Shuangying Cao, Min Yin, Peng Wang, Guqiao Ding, Liyou Yang, Jinrong Cheng, Linfeng Lu

**Affiliations:** 1 School of Materials Science and Engineering, Shanghai University, Shanghai, 200444 People’s Republic of China; 2 CAS Key Lab of Low-Carbon Conversion Science and Engineering, Shanghai Advanced Research Institute, Chinese Academy of Sciences, Shanghai, 201210 People’s Republic of China; 3 Center for Excellence in Superconducting Electronics (CENSE), State Key Laboratory of Functional Materials for Informatics, Shanghai Institute of Microsystem and Information Technology (SIMIT), Chinese Academy of Sciences, Shanghai, 200050 People’s Republic of China

**Keywords:** Graphene paper, Thin-film solar cells, Weight-specific power density, Flexibility

## Abstract

Flexible thin-film solar cells with high weight-specific power density are highly desired in the emerging portable/wearable electronic devices, solar-powered vehicles, etc. The conventional flexible metallic or plastic substrates are encountered either overweight or thermal and mechanical mismatch with deposited films. In this work, we proposed a novel substrate for flexible solar cells based on graphene paper, which possesses the advantages of being lightweight and having a high-temperature tolerance and high mechanical flexibility. Thin-film amorphous silicon (*a*-Si:H) solar cells were constructed on such graphene paper, whose power density is 4.5 times higher than that on plastic polyimide substrates. In addition, the *a*-Si:H solar cells present notable flexibility whose power conversion efficiencies show little degradation when the solar cells are bent to a radius as small as 14 mm for more than 100 times. The application of this unique flexible substrate can be extended to CuInGaSe and CdTe solar cells and other thin-film devices requiring high-temperature processing.

## Introduction

Mechanically flexible and lightweight thin-film solar cells can be attached to objects with curved surfaces, making them suitable as a source of electricity supply units for portable/wearable electronic devices and unmanned aerial vehicles [1–5]. By combining micro-electromechanical systems (MEMS) and bulk crystalline silicon solar fabricating technology, crystalline silicon solar cells with several micrometers thickness had been demonstrated with excellent flexibility [6]. Alternatively, flexible solar cells can also be realized by depositing absorbing layers together with other functional layers onto foreign substrates such as metallic [7–10] or plastic foils [11–14].

Because of the superior thermal stability and coefficient of thermal expansion (CTE), metallic foils are widely used as substrates for flexible solar cells [7, 8, 15–17]. The plastics possess better flexibility as well as lightweight characteristics. However, they usually have a low melting/softening temperature, which limits the processing temperature of solar cells (such as copper indium gallium selenide (CIGS)) that are typically accomplished under a high temperature [18–20]. The high CTE of the plastics may also induce the stress and strain accumulation in the thin films and lead to device failure or fast performance degradation. Among the plastic substrates, polyimide (PI) has a higher yield strain and lower density (1.4 g/cm^3^ vs 7.9 g/cm^3^ of stainless steel) [21, 22]. However, the thermal cycle process will induce a stress and strain accumulation due to the large mismatches of coefficient of thermal expansion (CTE) between PI material (12–40 10^−6^/K) [23, 24] and other inorganic layers, leading to macroscopic cracks and performance degradation [11, 25]. Cellulose paper also had been used to fabricate a-S:H solar cells, whose worse performance was also probably mainly due to the thermal expansion mismatch between the substrate and active layers [26]. Our recent work indicated that the construction of nanotextures on PI substrates can efficiently improve overall adhesion between atop films and the substrate and simultaneously release the internal thermal strain/stress [11, 13]. However, a tradeoff between mechanical compliance, performance, and robustness of flexible photovoltaic cells still remains as a major challenge.

Graphene, with many unique properties such as high strength and electrical and heat conductivity [27–30], has been wildly used in a variety of functional devices [31–34]. Recently, researchers have proposed a method that epitaxially grows high-quality materials and transfers them onto foreign substrates using single-layer graphene [35]. However, this transfer technology requires careful handling and complex processes, which is time-consuming and not compatible with large-scale production strategies.

As a derivate of graphene, graphene papers have been demonstrated by the solution-phase assembly, electrophoretic deposition, and chemical vapor deposition [27]. The excellent characteristics of high-temperature tolerance, low CTE, and mechanical flexibility would make it an ideal substrate for flexible electronics, especially, which will experience high-temperature processes [36, 37]. Among these researches, thin-film solar cells on graphene papers were seldom reported. In this work, we demonstrated flexible thin-film amorphous silicon (*a*-Si:H) solar cells on smooth graphene papers which were achieved by a filtration method using porous anodic aluminum oxide (AAO) filter. The device depicts a distinct weight-specific power density of 8.31 kW/kg, which is 415 and 4.5 times higher than the previous reports on glass and PI substrates, respectively [13, 38]. Moreover, the substrates endow the devices an outstanding bendable ability that the conversion efficiency only shows little degradation after 100 bending cycles with a radius as small as 14 mm. To the best of our knowledge, it is the first demonstration of thin-film solar cells on a graphene paper substrate. Although *a*-Si:H is used as the model material in this work with the overall processing temperature below 250 °C, the graphene paper substrates can be extended to other flexible (opto-)electronics, especially suitable for the devices requiring high-temperature processing.

## Materials and Methods

### Preparation of Graphene Papers

**Fig. 1:**
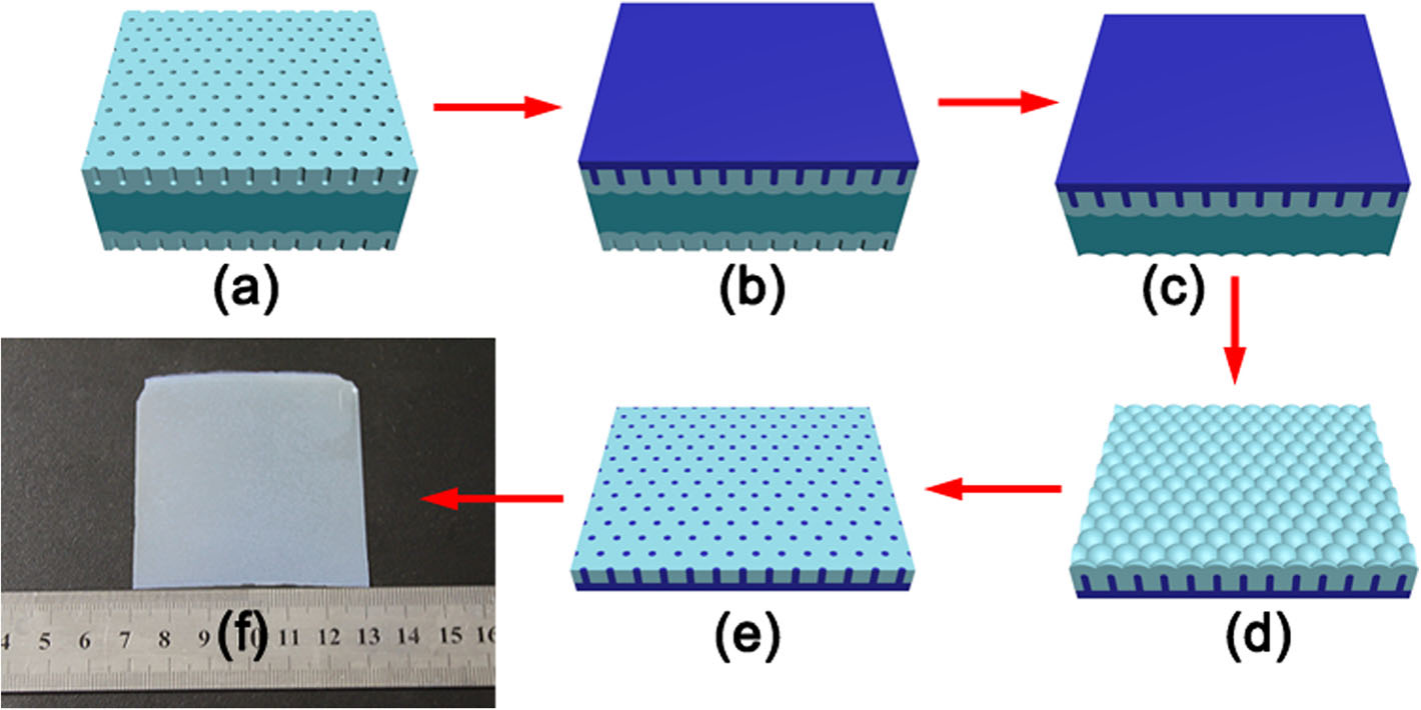
The fabrication processes of through-pore AAO filter membrane. (**a**) The as-obtained double side AAO on the Al foil. (**b**) Spin-coating a PMMA thin film on the one side. (**c**) Etching off AAO on the back side. (**d**) Removing the Al foil. (**e**) Dissolving the barrier layer in the AAO. (f) Removing the PMMA in glacial acetic acid and obtaining through-pore AAO filter membrane

The graphene papers were fabricated by solution-phase assembly procedure using vacuum filtration [27]. The filtration membrane is a through-pore AAO template prepared by ourselves using the procedure schematically illustrated in Fig. 1. The raw aluminum foils (99.999% purity) with typical dimensions of 70 mm × 60 mm × 0.3 mm were electropolished in a mixture of perchloric acid and ethanol (1:3 in volume) after ultrasonically cleaning in acetone, ethanol, and deionized water. After electropolishing, an anodization process was conducted in 0.3 M oxalic acid under a constant potential of 60 V at constant temperature 5 °C for 24 h (Fig. 1a). A polymethylmethacrylate (PMMA) film as a protective coating was firstly coated on one side of the double-sided anodized Al foil (Fig. 1b). The Al foil was immersed in 1 M NaOH to dissolve the AAO on the back side and obtain the one-side anodized Al foil (Fig. 1c). And then, it was immersed in a mixture containing 100 ml HCl, 3.7 g CuCl_2_·2H_2_O, and 100 ml deionized water to remove the remaining aluminum substrate and achieve the AAO film supported by PMMA (Fig. 1d). In order to fabricate through-hole AAO membranes, alumina barrier layer at the bottom of pores was chemically etched away in 5 wt% H_3_PO_4_ solution at 53 °C for 10 min (Fig. 1e). After etching in glacial acetic acid, the PMMA protective film was removed, resulting in a self-supporting through-hole AAO membrane. Finally, in order to increase the filtration capacity of AAO membrane, it was placed in 5 wt% H_3_PO_4_ solution for 20 min at 53 °C for a pore-opening process. The obtained through-pore AAO filter was a white, smooth sheet-like film, as shown in Fig. 1 f.

**Fig. 2:**
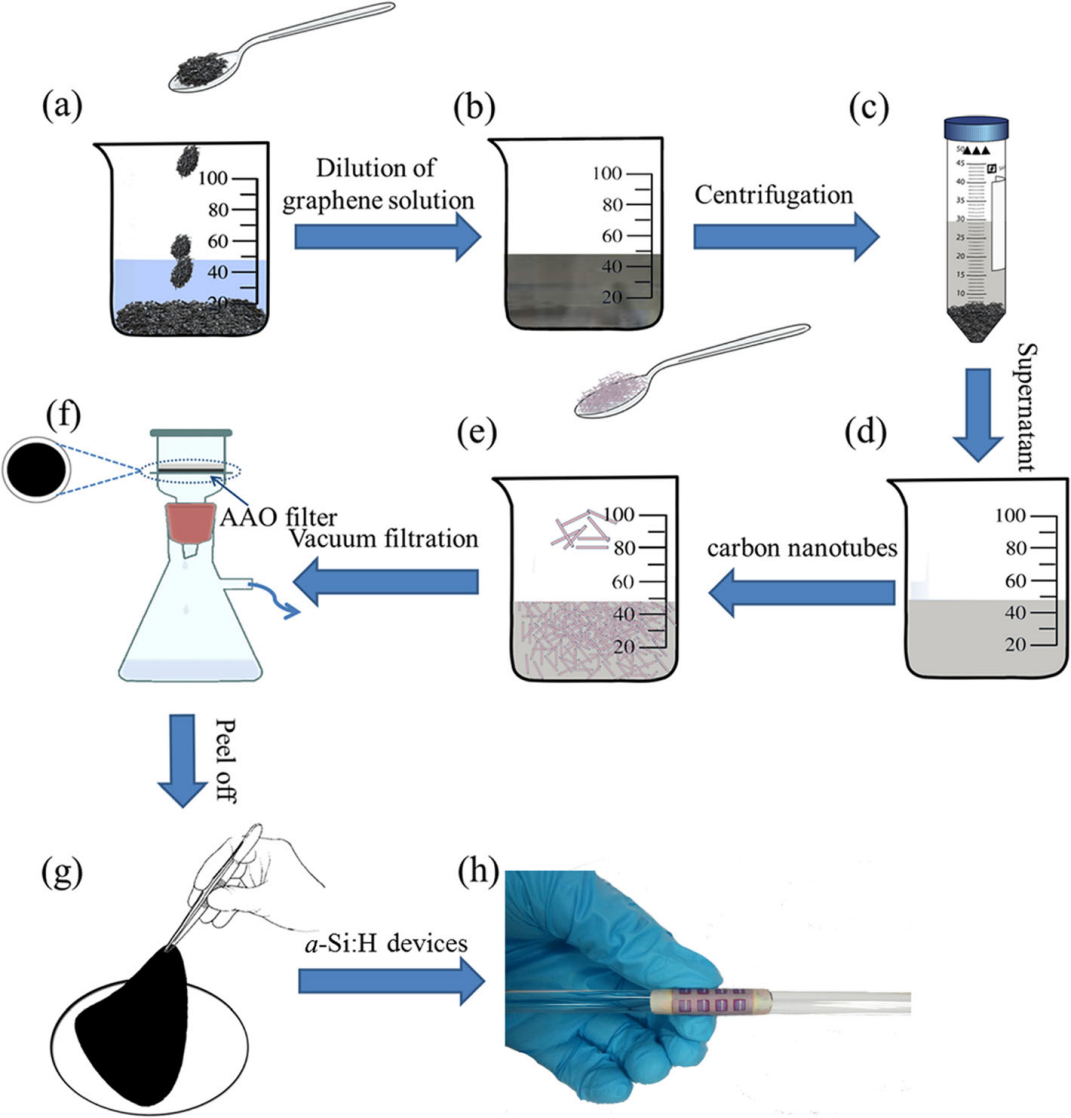
Fabrication procedures of the solar cell based on graphene paper. (**a**) Addition of reduced graphene oxide sheet in the aqueous solution of cetyltrimethylammonium bromide. (**b**) Dispersion of the mixture solution. (**c**) Centrifugation of the graphene solution. (**d**) Collection of the supernatant with well dispersed graphene flakes. (**e**) Addition of carbon nanotubes to the supernatant. (**f**) Vaccum filtration of the solutions over the through-hole anodic aluminum oxide membrane to obtain graphene paper on the anodic aluminum oxide filter. (**g**) Separation of graphene paper from the anodic aluminum oxide filter after drying. (**h**) a-Si:H solar cells fabricated on the graphene paper substrate demonstrate excellent flexibility by wrapping around the glass rod

The fabrication procedures of the solar cell based on graphene paper are schematically illustrated in Fig. 2. Firstly, 175 mg of cetyltrimethylammonium bromide (CTAB) as a stabilizer was dissolved in 500 ml of deionized water. Then, 250 mg of reduced graphene oxide sheet (Shanghai SIMBATT Energy Technology Co., Ltd.) was dispersed in the aqueous solution of CTAB (Fig. 2a). After that, the mixture solution was successively dispersed by an ultrasonic cleaner and cell disruptor for 1 h, respectively (Fig. 2b). After standing for 12 h, the graphene solution was centrifuged at 4500 rpm for 20 min to precipitate large particles (Fig. 2c) and leave supernatant with well-dispersed graphene flakes (Fig. 2d). As a comparison, graphene paper was also fabricated by using the original graphene solution without the centrifugal process. The graphene paper was then obtained by vacuum filtration (− 0.4 bar) of the solutions over the through-hole AAO membrane (Fig. 2f). The negative pressure was kept to ensure that the graphene film was always in close contact with the AAO filter during the drying process. After the drying process, graphene paper can be easily peeled off from the AAO filter which can be reused (Fig. 2g). The graphene papers which are defined as GP-1 (with the centrifugal process) and GP-2 (without the centrifugal process). Based on the same vacuum filtration, drying, and separation processes, the third sample, named as GP-3, was also prepared. GP-3 was obtained by adding a small amount of 10 wt% carbon nanotubes (CNTs) (10–20 nm in diameter, 5–15 μm length, Shenzhen Nanotech Port Co., Ltd) into the supernatant (Fig. 2e). Post annealing treatments at 400 °C for 1 h in argon atmosphere were also performed on all graphene papers in order to remove the residual solvent and surfactant.

### Preparation of Thin-Film *a*-Si:H Solar Cells

The fabrication of thin-film *a*-Si:H solar cells starts with sputtering of a 100-nm Ag layer on the graphene paper substrates, which serves as a back reflector. A 30-nm Al_2_O_3_-doped ZnO (AZO) layer as a spacer layer was then deposited by radio frequency (RF) magnetron sputtering of a 2 wt% AZO ceramic target (99.99% purity) at 250 °C. Subsequently, the *n*-*i*-*p a*-Si:H layers were deposited in a multi-chamber plasma-enhanced chemical vapor deposition (PECVD) system under 250 °C. The *n*, *i* and *p*-type layers were 30-, 280-, and 10-nm thick, respectively. After sputtering an 80-nm indium tin oxide (ITO) thin film, Ag grids were thermally evaporated as the top electrode using a contact mask (Fig. 2h) [6, 22]. For comparison, the solar cells were also fabricated on a glass substrate (1-mm thick, CSG Holding Co., Ltd.) under the same process. Other details of the preparation of *a*-Si:H solar cells can be found in our previous publications [7, 10, 11, 13, 39].

### Characterization

The surface morphologies were characterized by Hitachi S4800 scanning electron microscope (SEM). All the current density-voltage (*J-V*) curves of thin-film *a*-Si:H solar cells were carried out under 25 °C using a Xe lamp solar simulator (Newport, 94063A-1000, 100 mW/cm^2^) coupled with an air mass 1.5 global (AM 1.5 G) filter, and the external quantum efficiency (EQE) measurements were characterized by a commercial spectral response system (PV Measurement Inc. QEX10). The thermal stability of the graphene paper substrate was monitored by thermal gravimetry (TG) on a TG instrument (SDTA851 Switzerland-Mettler Toledo) from room temperature up to 1000 °C at a heating rate of 10 K/min. The reliability of solar cells under multiple bending cycles was performed with a home-built automatic bending setup [7, 11, 13].

## Results and Discussion

**Fig. 3:**
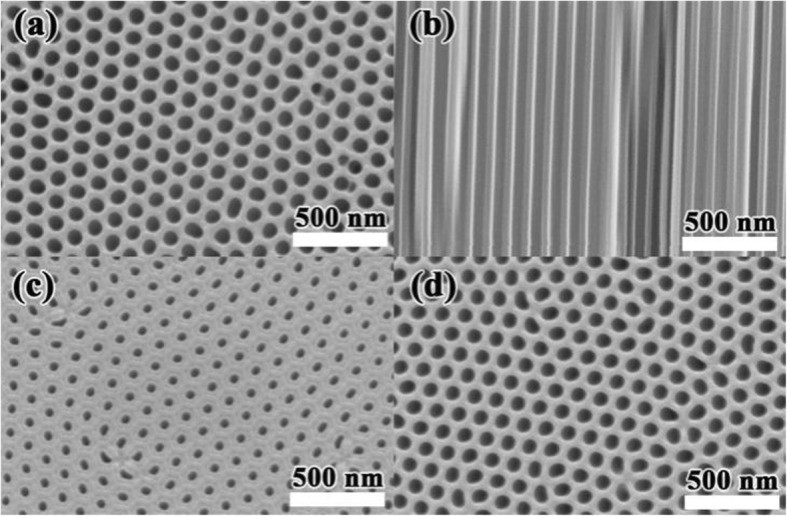
SEM images of **a** the surface, **b** cross-sectional view of the AAO membrane, and the bottom-view of the AAO membrane after etching the barrier Al layer for **c** 10 min, **d** 20 min

Figures 3 a and b show the surface and cross-sectional SEM images of the AAO membranes, respectively. The size of regular and uniform distributed holes is about 100 nm in diameter. The side walls of the AAO are smooth, which is a benefit for the filtration of the graphene solution. When the etching time is 10 min, there are residual barrier Al at the bottom of the AAO membrane, as shown in Fig. 3 c, leading to a hole size about 50 nm in diameter which is smaller than that of the front side. By extending the etching time to 20 min, the barrier oxide layer will be completely removed, resulting in 100 nm in diameter holes, same as the front side. Then, this AAO through-holes membrane with 20-min etching time is used for the filtration of graphene solution.

**Fig. 4:**
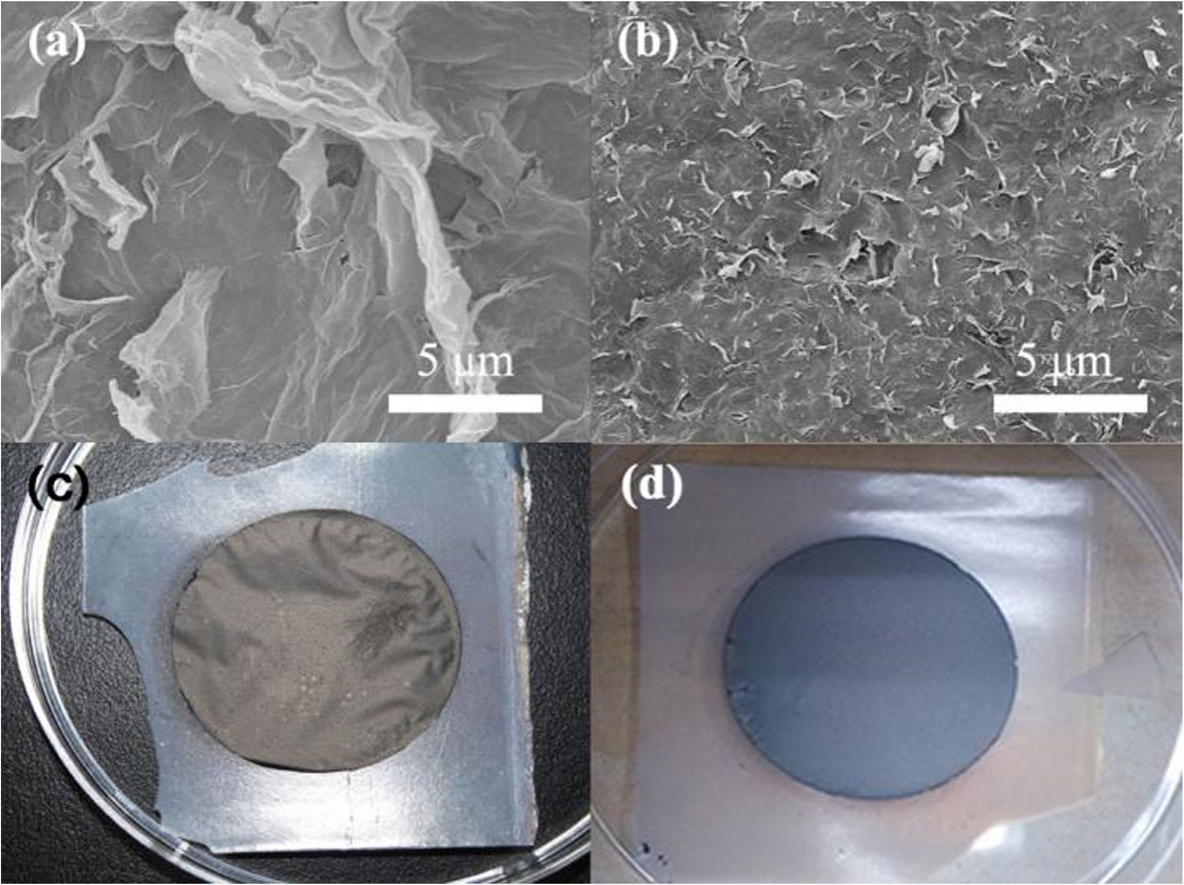
**a**, **c** SEM images and **b**, **d** digital camera images of (a, b) GP-1 and (b, d) GP-2

Figure 4 shows the SEM images and photographs of the GP-1 and GP-2 graphene papers. It is seen that the GP-1 (Fig. 4a) has a microscopic rough surface, which may be caused by the large-size graphene sheet and graphene clusters. These microscopic characteristics result in a macroscopic wrinkly surface as shown in Fig. 4 c. Due to the rough surface construction, pinholes and cracks can be easily formed in the following thin-film deposition. Therefore, high device performance can be hardly realized on the GP-1 graphene paper substrate.

The surface microstructure and corresponding photograph of GP-2 in Figs. 4 b and d indicate that the removal of precipitation of the graphene cluster is helpful to achieve a smooth surface. Unfortunately, although the smoothness of the GP-2 substrate has been improved, the mechanical strength of the GP-2 is not enough to withstand the bending experiment. After bending several times, *a*-Si:H solar cell on the GP-2 substrate will be cracked.

**Fig. 5:**
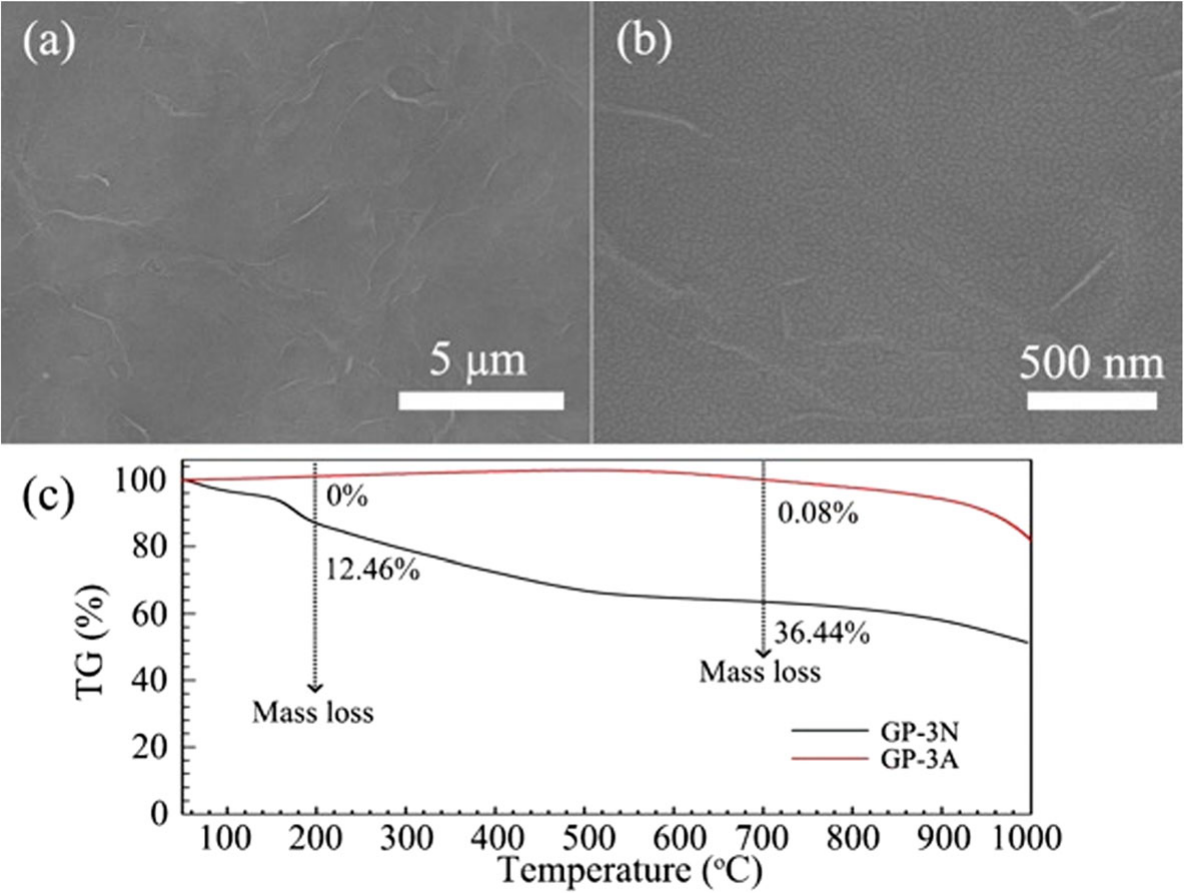
**a**, **b** SEM images of GP-3 graphene paper substrate with different magnifications. **c** The TGA results of the GP-3 graphene papers with (GP-3A) and without (GP-3N) post annealing treatment

To further enhance the mechanical strength of the graphene papers, CNTs are added to the supernatant of the graphene solution. The CNTs, serving as a mesh skeleton, would support the graphene sheet and in turn result in a better mechanical strength. Results from the bending experiment demonstrate that *a*-Si:H solar cells on GP-3 substrate have excellent flexibility which will be discussed later. Besides the improved mechanical strength, it is also found that the CNTs can effectively reduce the surface roughness in which the quite smooth morphology surface can be clearly observed as shown in the SEM images of Figs. 5 a and b. The smooth surface of graphene paper should be attributed to a layer of mesh skeleton composed of the carbon nanotubes because the graphene could enclose it [40]. This nanosized roughness is highly compatible with the following solar cell processes with respect to the high-quality and uniform thin-film layers.

The thermal stability of the GP-3 papers is investigated by thermo-gravimetric analysis (TGA) as a function of annealing process (Fig. 5c), where the papers without and with thermal processing (400 °C, 1 h, argon atmosphere) are denoted as GP-3N and GP-3A, respectively. An obvious weightlessness peak below 200 °C for the GP-3N paper indicates the dehydration of the crystallized water, which is accompanied by a mass loss of 12.46 %. As the temperature rises, the GP-3N paper continues to lose its mass. At 700 °C, a further loss of 23.98% can be observed, possibly due to the pyrolysis of unstable oxygen-containing functional groups [41]. For the sample that experienced post annealing process (GP-3A), the corresponding endothermic peak and weight loss can be hardly detected below 200 °C. Moreover, the GP-3A is thermally stable up to 700 °C with only a 0.08% weight loss. These results imply that the moisture and the thermally labile oxygen functional groups have been efficiently removed during the annealing treatment [42].

**Fig. 6:**
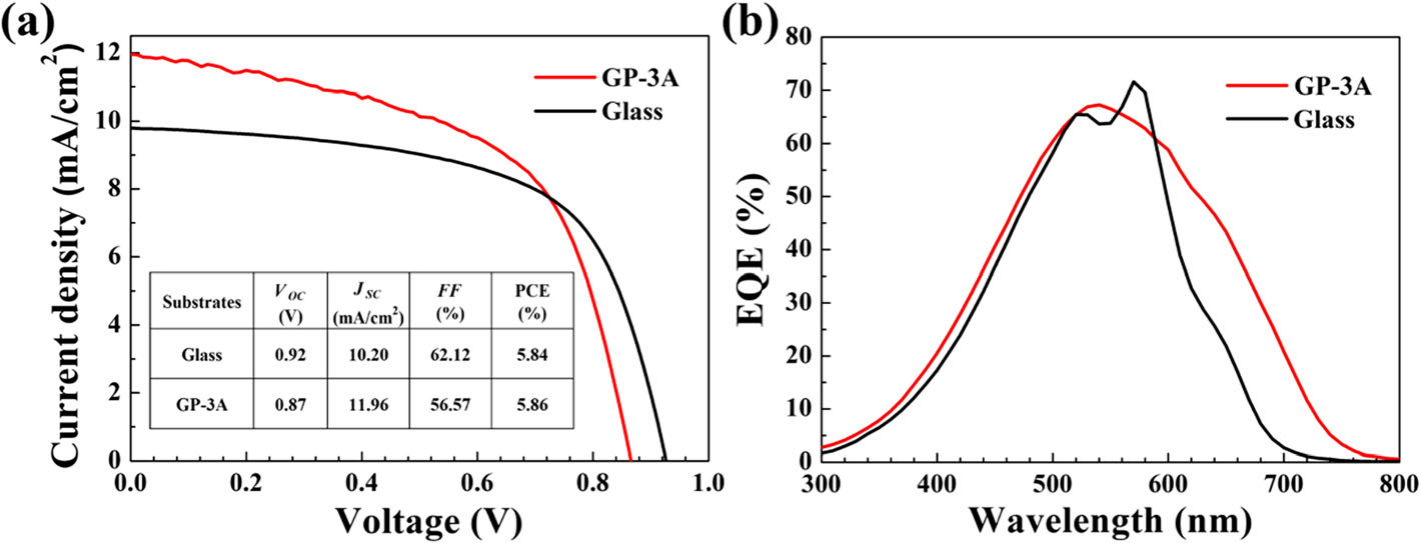
**a** *J*-*V* curves and **b** EQE measurements of *a*-Si:H devices based on the GP-3 and glass substrates

Due to the improved mechanical durability and surface roughness, the GP-3A papers are chosen as the substrates for the fabrication of *a*-Si:H solar cells. Its thickness and weight are 53 μm and 5.73 mg, respectively. The device deposited on a rigid glass substrate is also fabricated as a reference. Figure 6 a shows the current density-voltage (*J-V*) characteristics of the devices on both GP-3A and glass substrates measured under AM 1.5-G irradiation. A power conversion efficiency (PCE) of 5.86% is obtained on the GP-3A substrate, with an open-circuit voltage (*V*_OC_) of 0.87 V, a short circuit current (*J*_SC_) of 11.96 mA/cm^2^, and a fill factor (*FF*) of 0.57. Compared with the device on glass substrate, the *J*sc is improved by 17%, which is further confirmed by EQE measurements (Fig. 6b). The GP-3A substrate renders a broadband spectral response enhancement especially in the long-wavelength range above 600 nm.

The improved spectral response may be ascribed to the small wrinkles on the graphene paper, which increase the effective optical path by scattering the long-wavelength light at the device bottom. However, the surface defects may induce shunt channels for the current that results in the decrease of *V*_OC_ and *FF*. In addition, the trapped gas in the graphene paper may experience expansion-inducing stresses on the contact layers, which could be also responsible for the decrease of *V*_*OC*_ and *FF*. Therefore, although the photocurrent of the device on GP-3A increases significantly, the final energy conversion efficiency is not dramatically improved compared to that of the solar cells on glass substrate.

As the thickness of *a*-Si:H solar cells are only a few hundred nanometers, the substrates will dominate the weight and thickness of the ultimate devices. In this work, we demonstrated flexible solar cells on graphene papers which are much lighter than conventional glass and plastic substrates. Herein, we further compare the weight-specific power density (*P*_W_) of devices on different substrates. The *P*_W_ is defined as the ratio of the output power under standard solar irradiation (AM 1.5 Global spectrum with 1000 W m^−2^ intensity) to the mass of the solar cell per unit area as expressed by following equation:

1 $$ {P}_{\mathrm{W}}=\left(1000\ \mathrm{W}{\mathrm{m}}^{-2}\times \mathrm{PCE}\right)/{m}_{\mathrm{d}} $$

where the *m*_d_ is the mass of the solar cell per unit area.

Compared with the devices deposited on the glass substrate, the thickness and weight of solar cells on graphene papers are reduced by ~ 20 times and ~ 350 times, respectively. Meanwhile, the power density reaches as high as 8.31 kW/kg, which is 415 times higher than that of its counterpart. In our previous work, *a*-Si:H solar cells were successfully fabricated on the patterned PI flexible substrates [13]. The GP-3A device has lower energy conversion efficiency than the devices on PI substrates because of the absence of period back reflectors on GP-3A substrate, while the solar cells on the weight of graphene papers are only 15% of the PI substrate. Therefore, the power density of the GP-3A device is 4.52 times higher than that on patterned PI substrate. And the details of characteristic parameters of *a*-Si:H solar cells based on GP-3A and the counterpart substrates are summarized in Table 1.

**Table 1:** Summary of power conversion efficiency (PCE), thickness and weight-specific power of *a*-Si:H solar cells based on GP-3A, glass and PI substrates

	PCE (%)	Thickness (μm)	Weight-specific power density (kW/kg)
GP-3A	5.86	53	8.31
Glass	5.84	1000	0.02
PI	7.71 [13]	25	1.84

**Fig. 7:**
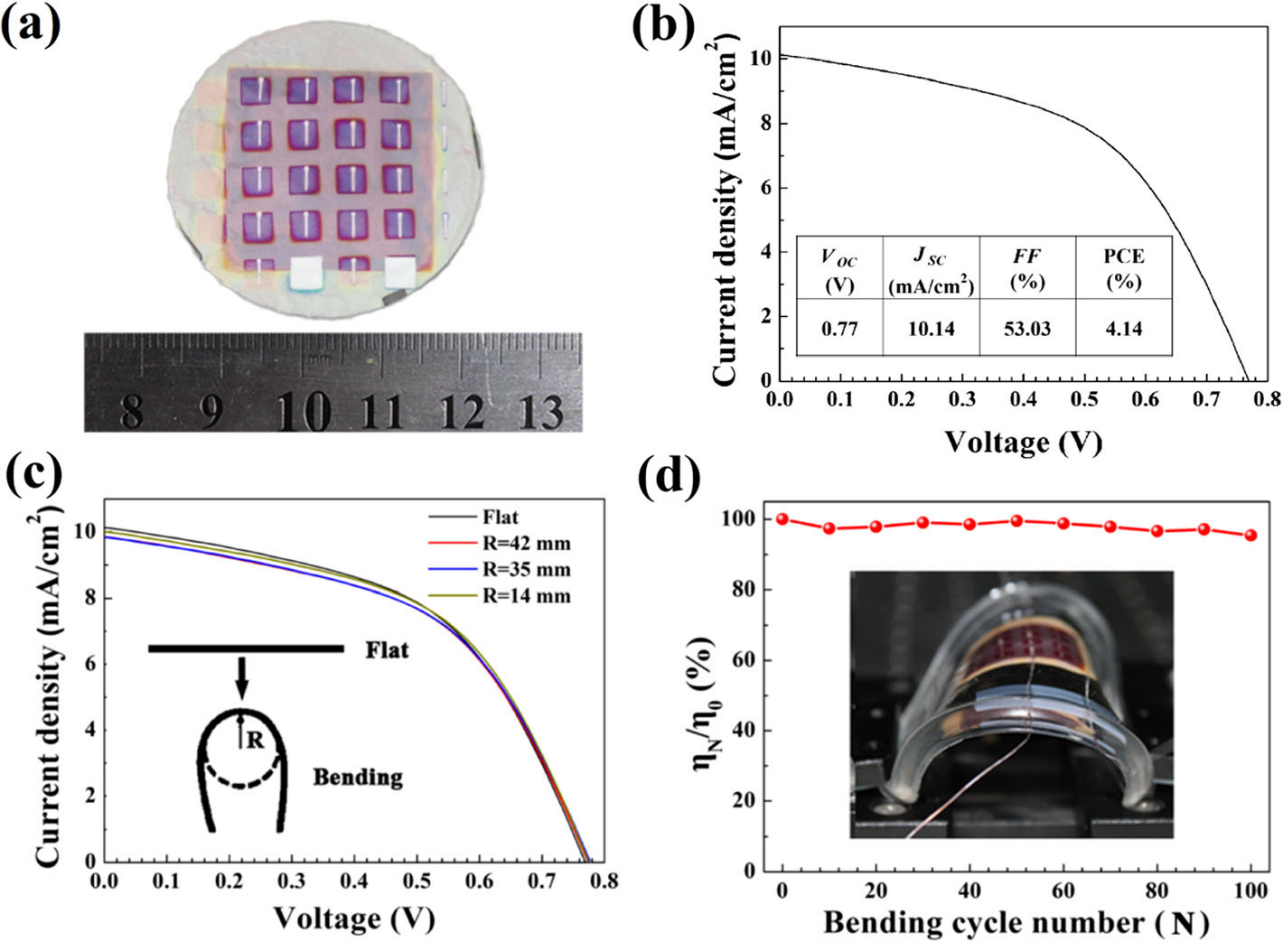
**a** A photograph of the *a*-Si:H solar cells on the GP-3A. **b** A *J-V* curves of *a*-Si:H devices based on the GP-3A substrate after encapsulation. **c** *J*-*V* curves of a device on GP-3A substrate under different bending angles. **d** Relative efficiency as a function of bending cycles. The insets in **c** and **d** represent an illustration of defining bending angle and a bended device mounted on the measurement setup, respectively

Graphene paper, which possesses superior flexibility, is lightweight, and has high-temperature tolerance, is expected to be an alternative choice for portable device application. Figure 7 a shows a picture of the actual devices obtained on a graphene paper. In order to evaluate the durability of graphene paper-based devices during flexible operation, the solar cells are then encapsulated by polydimethylsiloxane (PDMS) and its electrical contacts were made by copper wires. Figure 7 b shows the measured *J-V* curves of the *a*-Si:H devices after encapsulation. Unfortunately, the energy conversion efficiency of the device was decreased from 5.86 down to 4.14% after the encapsulation. It may be because the copper wire electrodes generate additional contact resistance and slightly damage the device. Thus, elaborate encapsulation strategies would be developed for such GP-based ultra-light devices in future work. After encapsulation, the *J-V* curves under various bending angles are characterized with a homemade setup [7]. The flexible performances are evaluated as a function of bending radius and bending cycle [7, 8, 13]. Figure 7c indicates that the cells on the GP-3A can endure the manual bending tests with a radius down to 14 mm and maintain full function. Moreover, the reliability of GP-3A solar cell under repeated bending (radius = 14 mm) is characterized as presented in Fig. 7 d, while the device still retained above 92% of the initial efficiency after 100 bending cycles. The outstanding flexibility and stability could dominantly benefit from the ultrathin graphene paper, as well as the higher mechanical strength of the graphene paper modified by CNTs.

## Conclusion

In this work, we developed a carbon nanotube-enhanced graphene paper substrate which delivered higher thermal stability, is lightweight, and has excellent mechanical flexibility over traditional flexible substrates. The *a*-Si:H solar cells based on graphene papers were successfully achieved with better photocurrents and comparative energy conversion efficiencies (5.86%) over the counterparts on flat glass substrates. The thickness and weight of solar cells on graphene paper are reduced by ~ 20 and ~ 350 times, respectively. Meanwhile, the power density reaches as high as 8.31 W/g, which is 415 times higher than that of the counterparts. Moreover, the devices based on graphene paper presented an excellent performance with a marginal drop even after 100 bending cycles under 14-mm radius due to ultrathin thickness and excellent mechanical flexibility of graphene paper substrates. Although the work was conducted on *a*-Si:H material, our proposed scheme can be extended to other material systems that may lead to a new era of flexible optoelectronic devices.

## Data Availability

The datasets generated during and/or analyzed during the current study are available from the corresponding authors on reasonable request.

## Abbreviations

AAO: Anodic aluminum oxide
*a*-Si:H: Amorphous silicon
AZO: Al_2_O_3_-doped ZnO
CIGS: Copper indium gallium selenide
CNTs: Carbon nanotubes
CTAB: Cetyltrimethylammonium bromide
CTE: Coefficient of thermal expansion
EQE: External quantum efficiency
*FF*: Fill factor
GP: Graphene paper
ITO: Indium tin oxide
*J*_SC_: Short circuit current
MEMS: Micro-electromechanical systems
PCE: Power conversion efficiency
PECVD: Plasma-enhanced chemical vapor deposition
PI: Polyimide
*P*_W_: Weight-specific power density
RF: Radio frequency
SEM: Scanning electron microscope
TGA: Thermo-gravimetric analysis
*V*_OC_: Open-circuit voltage
